# Complete Genome Sequences of Three Related Avian Avulavirus 1 Isolates from Poultry Farmers in Pakistan

**DOI:** 10.1128/genomeA.00361-18

**Published:** 2018-05-03

**Authors:** M. Zubair Shabbir, Ruth Helmus Nissly, Abdul Ahad, Masood Rabbani, Shubhada K. Chothe, Aswathy Sebastian, Istvan Albert, Bhushan M. Jayarao, Suresh V. Kuchipudi

**Affiliations:** a University of Veterinary and Animal Sciences, Lahore, Pakistan; b Animal Diagnostic Laboratory, Department of Veterinary and Biomedical Sciences, The Pennsylvania State University, University Park, Pennsylvania, USA; c Chittagong Veterinary and Animal Sciences University, Chittagong, Bangladesh; d Bioinformatics Consulting Center, The Pennsylvania State University, University Park, Pennsylvania, USA

## Abstract

Avian avulavirus 1 infects multiple avian hosts, and rare reports of human infection have been noted throughout the last century. Here, we report the complete genome sequences of three isolates of avulavirus 1 collected from poultry farmers in Pakistan exhibiting mild respiratory signs.

## GENOME ANNOUNCEMENT

Avian avulavirus 1 (AAvV-1) is a member of the genus Avulavirus of the family Paramyxoviridae (1). The AAvV-1 species includes multiple strains of avian paramyxovirus 1 (APMV-1) virus. APMV-1 strains have a genome size of around 15,000 nucleotides, consisting of negative-sense single-stranded RNA that encodes six structural proteins. APMV-1 is of particular importance to poultry due to virulent strains which cause Newcastle disease, often referred to as Newcastle disease viruses (NDVs) (2). At least two classes and 18 genotypes of APMV-1 have been described (3). In Pakistan, the prevalent virulent strains of NDV are classified within genotypes VI and VII; genotype VII is predominant across the country. Generally, APMV-1 infects avian species, but rare accounts have identified APMV-1 infection in humans (4), typically in poultry workers or immunocompromised patients. We report here the full-genome sequences of three AAvV-1 isolates recovered from human poultry workers showing mild respiratory symptoms.

From October 2010 to December 2011, nasal swabs were collected from three individual patients in the Lahore and Sheikhupura districts of Pakistan, with approval from the ethics research committee at the University of Veterinary and Animal Sciences, Lahore, Pakistan (Department of Pharmacology record number 1349, 19-09-2009). Using the Illumina TruSeq stranded mRNA kit, a barcoded library was prepared from each RNA sample. The manufacturer’s protocol was followed using the “purified mRNA input” recommendation, which skips poly(A) RNA enrichment and begins with RNA fragmentation and priming. An equimolar pool of libraries was made and sequenced on an Illumina MiSeq using 150 × 150-bp paired-end sequencing, according to the manufacturer’s protocol. Between 29,947 and 82,808 reads were assembled for each genome. Following FastQC, the paired-end reads were merged using FLASH (5) and then run against the NCBI nonredundant (nr)-protein database using DIAMOND (6). The results were visualized with MEGAN6 (7), revealing a close relationship to NDV. Reads belonging to all viruses were extracted, and the sequence was assembled using SPAdes (8). From the assembly, the largest contigs (>500 bases) were extracted, and these were used as a query for the BLAST search against the nr-genome database. The top hit was goose paramyxovirus SF02 (GenBank accession number NC_005036), and the second hit was Newcastle disease virus strain B1 (GenBank accession number NC_002617). Contigs were aligned against each of these reference strains separately. Nodes were reverse complemented as required and concatenated based on alignment with the goose paramyxovirus reference.

The viral genomes contained six transcriptional units (3′-NP-P-M-F-HN-L-5′). The encoded fusion (F) protein from all three isolates has the same amino acid sequence. The sequence of the cleavage activation site of the fusion protein is ^109^SGGRRQKR↓FIG^119^, consistent with velogenic strains of NDV. Phylogenetic analysis of complete fusion protein sequences indicated that the three new isolates were closely related to other velogenic Pakistani isolates of the genotype XIII, which was further supported by the presence of serine at amino acid position 107 (9, 10). Indeed, the cleavage site sequence was identical to chicken isolates from Munir et al. (11).

### Accession number(s).

The complete genome sequences of these AAvV-1 isolates have been deposited in GenBank under the accession numbers MH019281, MH019282, and MH019283.
